# Supplementary material for: Rapid assessment of the temporal function and phenotypic reversibility of neurodevelopmental disorder risk genes in Caenorhabditis elegans
Source: Dis Model Mech. 2022 May 6;15(5):dmm049359. doi: 10.1242/dmm.049359 (PMC9092656; doi:10.1242/dmm.049359)
Supplement: Supplementary information [file dmm-15-049359-s1.pdf]

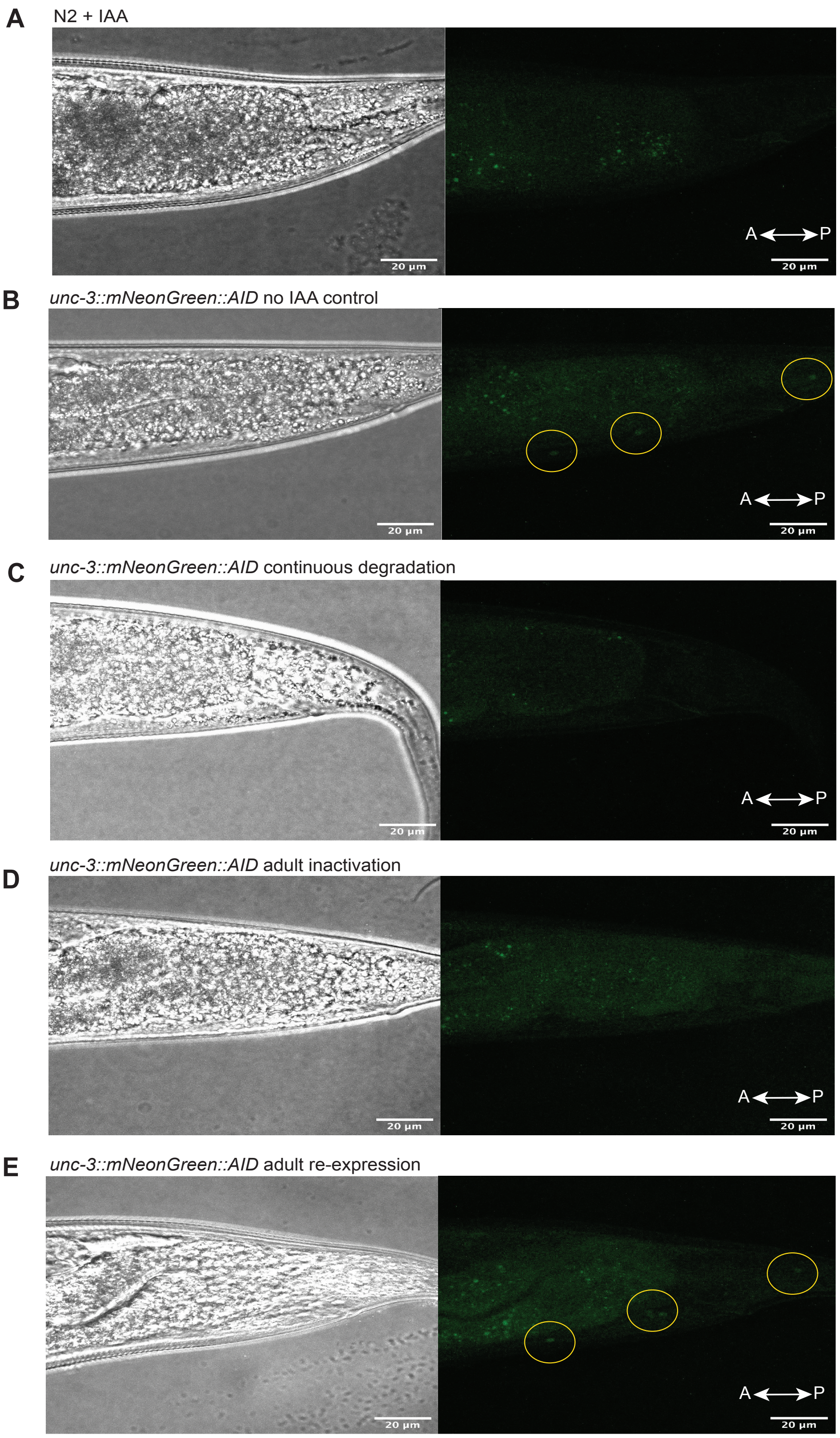

Fig. S1. Expression of *UNC-3::mNeonGreen::AID* across temporal degradation conditions. All images are taken when animals were 96hours old. A) N2 animal continuously grown in the presence of IAA. B) UNC-3::mNeonGreen::AID expression in the no-IAA control. Yellow circles highlight mNeonGreen fluorescence in neuron cell bodies in the *C. elegans* tail.

Disease Models & Mechanisms • Supplementary information

C) UNC-3::mNeonGreen::AID expression in animals continuously grown on IAA plates. No visible fluorescence was detected. D) UNC-3 expression in animals transferred to IAA plates at L4. There was no fluorescence visible, indicating efficient degradation of UNC-3 after 48 hours of IAA exposure. E) UNC-3::mNeonGreen::AID expression in animals taken off IAA plates at L4. mNeonGreen fluorescence was visible in neuron cell bodies (highlighted by yellow circles). Images representative of 4-5 animals scored for the presence of mNeonGreen.

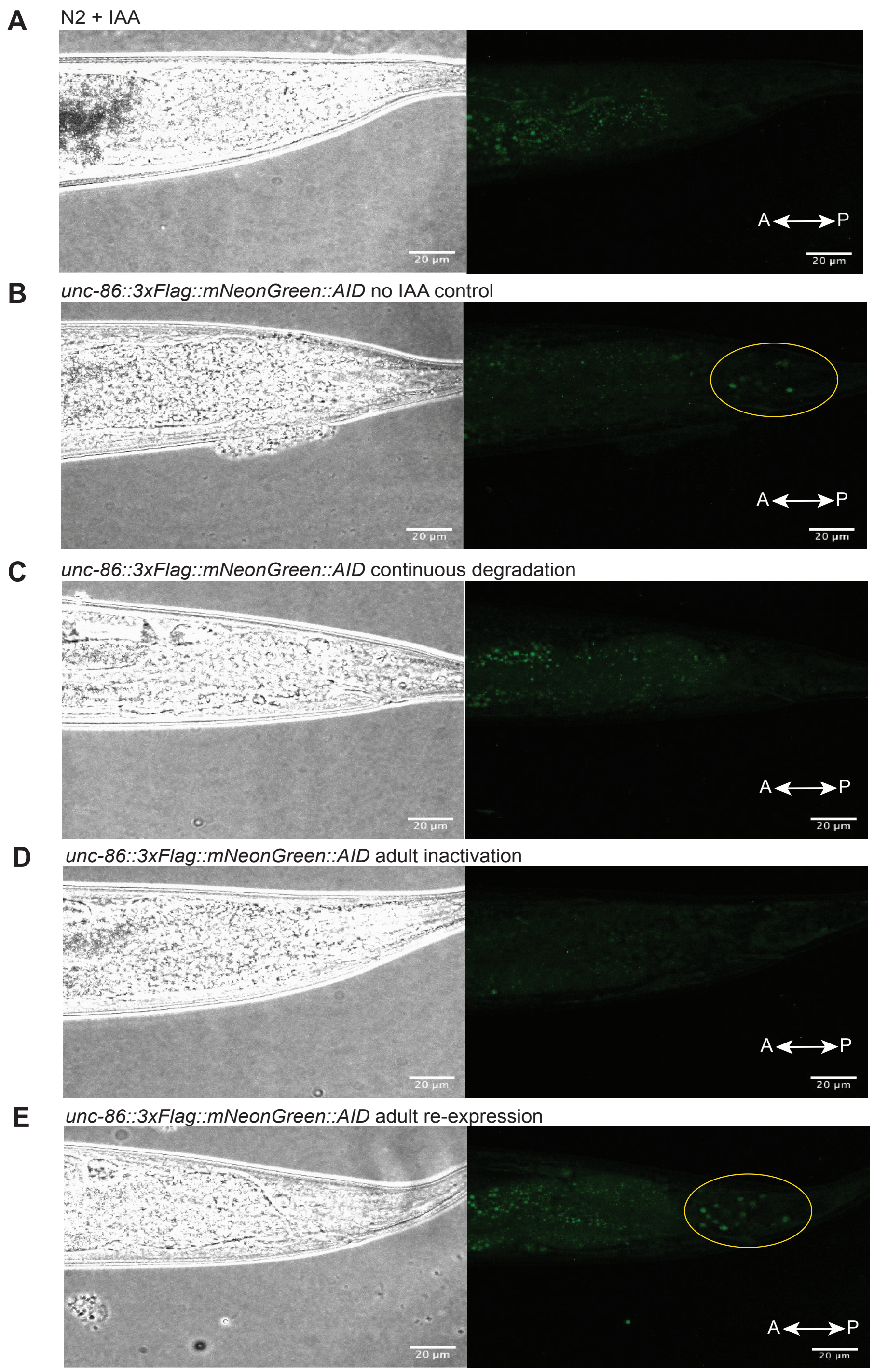

Fig. S2. Expression of *UNC-86::3xFlag::mNeonGreen::AID* across temporal degradation conditions. All images are taken when animals were 96hours old. A) N2 animal continuously grown in the presence of IAA. B) UNC-863xFlag::mNeonGreen::AID expression in the no-IAA control. Yellow circles highlight mNeonGreen fluorescence in neuron cell bodies in the *C. elegans* tail. C) UNC-863xFlag::mNeonGreen::AID expression in animals continuously

Disease Models & Mechanisms • Supplementary information

grown on IAA plates. No visible fluorescence was detected. D) UNC-86 expression in animals transferred to IAA plates at L4. There was no fluorescence visible, indicating efficient degradation of UNC-86 after 48 hours of IAA exposure. E) UNC-863xFlag::mNeonGreen::AID expression in animals taken off IAA plates at L4. mNeonGreen fluorescence was visible in neuron cell bodies (highlighted by yellow circles). Images representative of 4-5 animals scored for the presence of mNeonGreen.

Disease Models & Mechanisms • Supplementary information

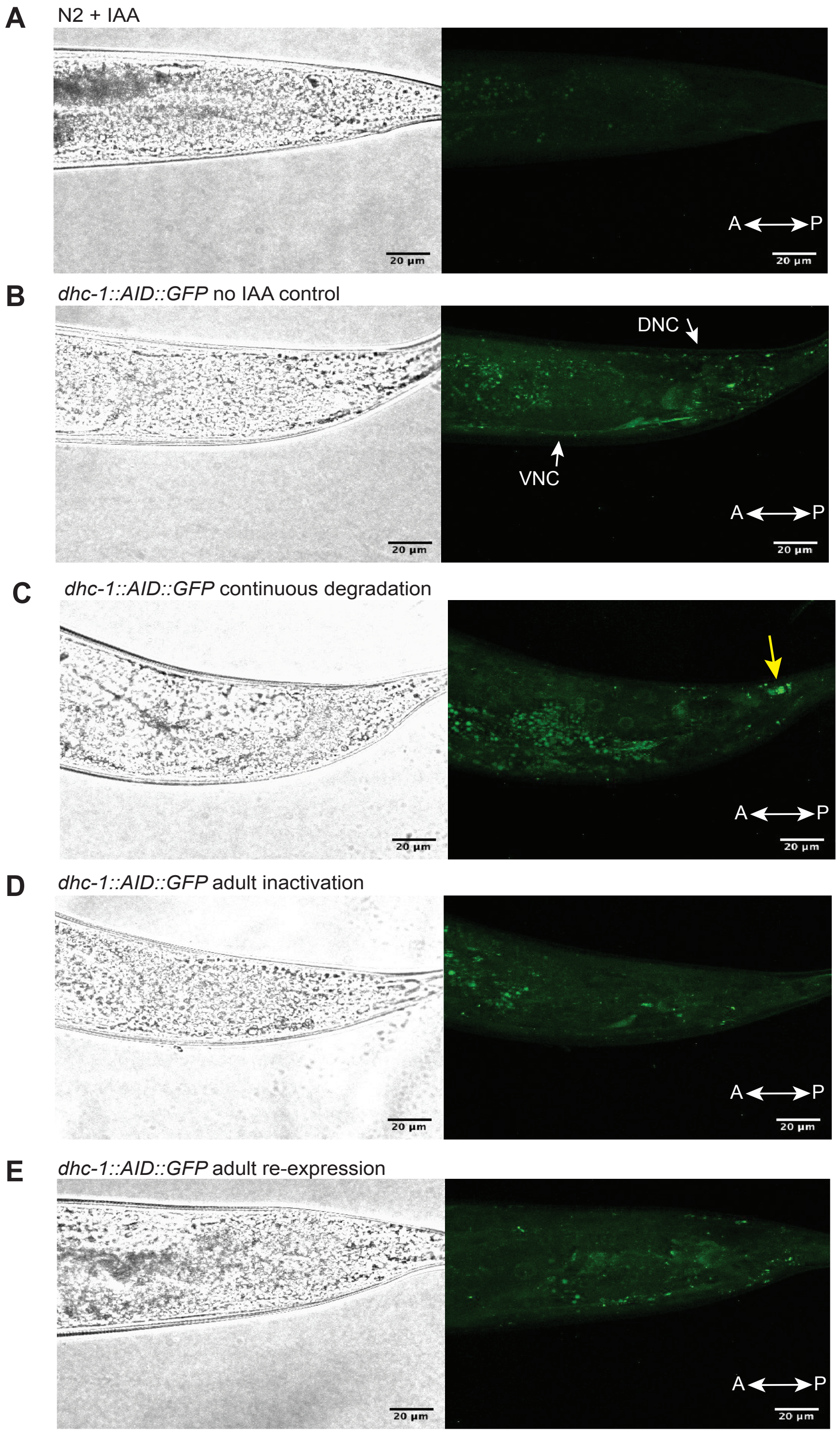

Fig. S3. Expression of *DHC-1::AID::GFP* in neuron-specific temporal degradation conditions. All images are taken when animals were 96hours old. A) N2 animal continuously grown in the presence of IAA. B) DHC-1::AID::GFP expression in the no-IAA control. Fluorescence was visible in the ventral nerve cord (VNC) and dorsal nerve cord (DNC). C)

Disease Models & Mechanisms • Supplementary information

DHC-1::AID::GFP expression in animals continuously exposed to IAA to induce protein degradation in neurons only. There was decreased fluorescence in the nerve cords, and abnormal aggregation on GFP in the tail (highlighted by yellow arrow). D) DHC-1::AID::GFP expression in animals transferred to IAA plates at L4 to induce adult degradation. There was decreased GFP fluorescence in the VNC and DNC. E) DHC-1::AID::GFP expression in animals taken off IAA plates at L4. GFP fluorescence was visible in the VNC and DNC. Images representative of 4-5 animals scored for the presence of GFP.

A

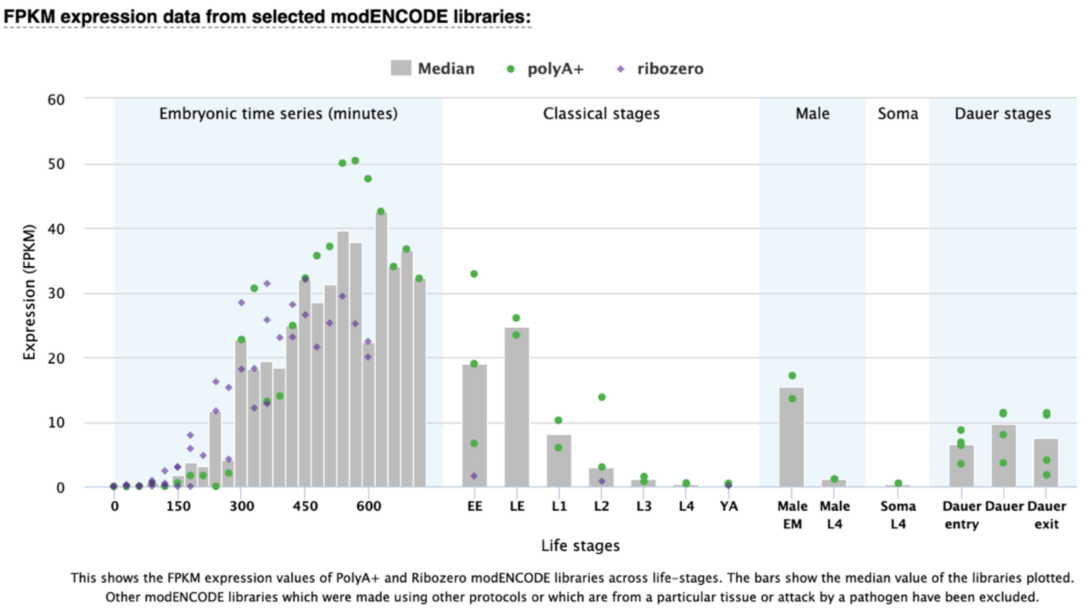

B

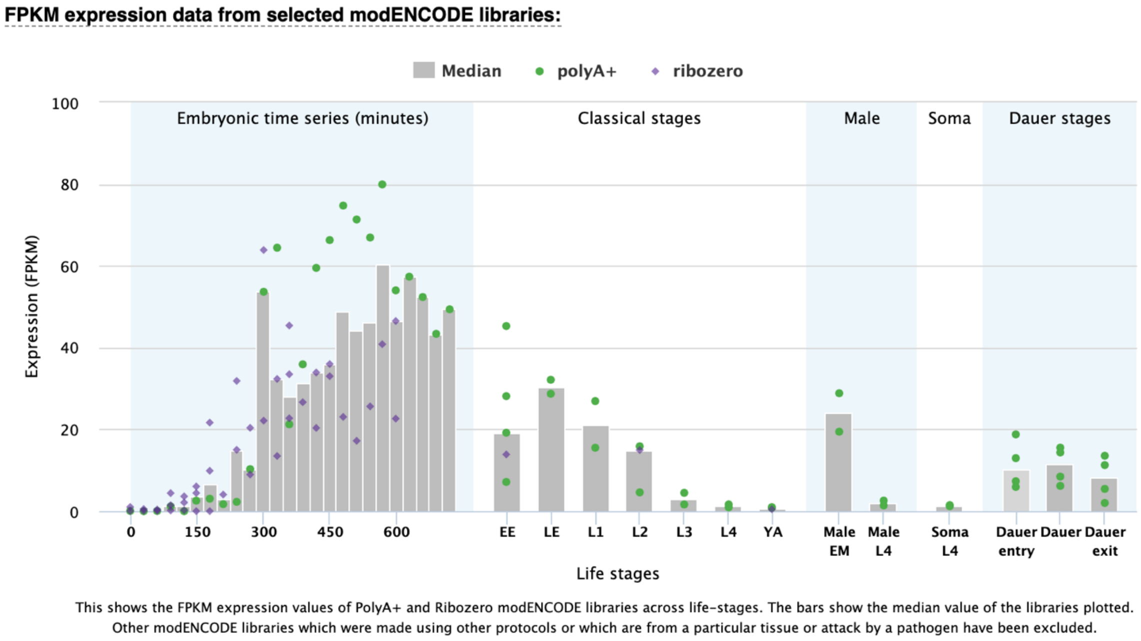

Fig. S4. *unc-3* and *unc-86* have a similar pattern of expression across the lifespan. A) Expression pattern of *unc-3* across the *C. elegans* lifespan. B) Expression pattern of *unc-86* across the *C. elegans* lifespan. Downloaded from Wormbase.org (Gerstein et al., 2010). Both transcription factors show high expression in embryonic and early larval development, with lower expression in late development and adulthood. FPKM (fragments per kilobase of transcript per million mapped reads).

Disease Models & Mechanisms • Supplementary information

Table S1. Phenotypic feature descriptions.

Feature	Description
Width	The distance spanned perpendicular to the major axis, in mm.
Length	The distance spanned by objects along their major axis (defined to be the axisof a least squares fit), in mm.
Morph Width	The mean width of the central 60% of the object, measured perpendicular to theskeleton. This is only available if the object has a skeleton.
Curve	The average angle, in radians, of points along the object's skeleton taken twoapart. This measure is only available if skeletons exist.
Area	The area in square millimeters of objects
Midline Length	The length of the object, in mm, measured along the skeleton; this is only available if skeletons were collected (or outlines were collected, and skeletonswere generated with a plugin).
Path Length	The distance that the animal has traveled. This is both signed (forwards is positive, backwards is negative) based on the results of the “bias” output andcumulative. If movement direction cannot be computed, then the length startsagain from zero.
Bias	The fraction of objects moving in the dominant movement direction as opposedto the other direction. The dominant direction is labeled 1, the opposite 1, and stationary objects are 0. This is usually inaccurate unless the segment (S) option is used.
Direction	The frequency of changes in direction. Forward motion is 1; motion that backtracks is 1 at the point of turning. This metric is somewhat unreliable unlesssegment (S) is used.
Angular Speed	The angular speed in radians/second of objects; this is calculated over the sameinterval as speed, but reports the greatest difference in angle between primary axes over that time
Aspect	The ratio of the width to length
Kink	The angle in radians between the line from the first to third point of the skeletonand the fourth through last points; or the angle between first and fourth to last and third to last to last, whichever is greater. This is similar to the MWT endwiggle measure.
Crab	The speed perpendicular to the length of the body, in mm/sec, averaged as specified by s. Note that the speed along the length of the body is sqrt (speed*speed – crab*crab).
	The speed in mm/second of objects. This speed is averaged over the duration given in the s parameter; it is equal to the distance between the

Disease Models & Mechanisms • Supplementary information

Baseline Speed	most widely spaced pair of points over that period of time (half ahead of and half behind thecurrent timepoint) divided by that duration.
Initial Reversal Response Probability	The proportion of animals reversing to the first mechanosensory stimulus.
Initial Reversal Response Duration	The duration of reversal responses to the first mechanosensory stimulus
Initial Reversal Response Speed	The speed of reversal responses to the first mechanosensory stimulus
Initial Reversal Response Distance	The distance (mm) of reversal responses to the first mechanosensory stimulus
Habituation of Response Probability	The difference between the initial reversal response probability and the final habituated level of response probability (average of 28-30th responses).
Habituation of Response Duration	The difference between the initial reversal response duration and the final habituated level of response duration (average of 28-30th responses).
Habituation of Response Speed	The difference between the initial reversal response speed and the final habituated level of response speed (average of 28-30th responses).
Habituation of Response Distance	The difference between the initial reversal response distance and the final habituated level of response distance (average of 28-30th responses).
Mean Response Probability	The mean reversal response probability across the habituation paradigm (average of all responses).
Mean Response Duration	The mean reversal response duration across the habituation paradigm (average of all responses).
Mean Response Speed	The mean reversal response speed across the habituation paradigm (average of all responses).
Mean Response Distance	The mean reversal response distance across the habituation paradigm (average of all responses).
Spontaneous Recovery of Response Probability	The reversal response probability to the 31st stimulus (delivered 5 minutes after the habituation session to gauge spontaneous recovery of habituation).
Spontaneous Recovery of Response Duration	The reversal response duration to the 31st stimulus (delivered 5 minutes after the habituation session to gauge spontaneous recovery of habituation).

Disease Models & Mechanisms • Supplementary information

Spontaneous Recovery of Response Speed	The reversal response speed to the 31st stimulus (delivered 5 minutes after the habituation session to gauge spontaneous recovery of habituation).
Spontaneous Recovery of Response Distance	The reversal response distance to the 31st stimulus (delivered 5 minutes after the habituation session to gauge spontaneous recovery of habituation).

Disease Models & Mechanisms • Supplementary information
